# Topological surface states of semimetal TaSb_2_

**DOI:** 10.1186/s40580-024-00457-y

**Published:** 2024-12-02

**Authors:** Ji-Eun Lee, Yu Liu, Jinwoong Hwang, Choongyu Hwang, Cedomir Petrovic, Se Young Park, Hyejin Ryu, Sung-Kwan Mo

**Affiliations:** 1 Advanced Light Source, Lawrence Berkeley National Laboratory, Berkeley, CA 94720 USA; 2 Center for Spintronics, Korea Institute of Science and Technology (KIST), Seoul, 02792 South Korea; 3 Max Planck POSTECH Center for Complex Phase Materials, Pohang University of Science and Technology, Pohang, 37673 South Korea; 4 Center for Correlated Matter and School of Physics, Zhejiang University, Hangzhou, 310058 China; 5 Condensed Matter Physics and Materials Science Department, Brookhaven National Laboratory, Upton, NY 11973 USA; 6 Department of Physics and Institute of Quantum Convergence Technology, Kangwon National University, Chuncheon, 24341 South Korea; 7 Department of Physics, Pusan National University, Busan, 46241 South Korea; 8 Shanghai Key Laboratory of Material Frontiers Research in Extreme Environments (MFree), Shanghai Advanced Research in Physical Sciences (SHARPS), Pudong, Shanghai, 201203 China; 9 Department of Physics and Origin of Matter and Evolution of Galaxies (OMEG) Institute, Soongsil University, Seoul, 06978 South Korea; 10 Integrative Institute of Basic Sciences, Soongsil University, Seoul, 06978 South Korea

**Keywords:** Topological materials, Electronic structures, TaSb_2_, Resistivity plateau, Extremely large magnetoresistance

## Abstract

Topological surface states, protected by the global symmetry of the materials, are the keys to understanding various novel electrical, magnetic, and optical properties. TaSb_2_ is a newly discovered topological material with unique transport phenomena, including negative magnetoresistance and resistivity plateau, whose microscopic understanding is yet to be reached. In this study, we investigate the electronic band structure of TaSb_2_ using angle-resolved photoemission spectroscopy and density functional theory. Our analyses reveal distinct bulk and surface states in TaSb_2_, providing direct evidence of its topological nature. Notably, surface states predominate the electronic contribution near the Fermi level, while bulk bands are mostly located at higher binding energies. Our study underlines the importance of systematic investigations into the electronic structures of topological materials, offering insights into their fundamental properties and potential applications in future technologies.

**Graphical Abstract:**

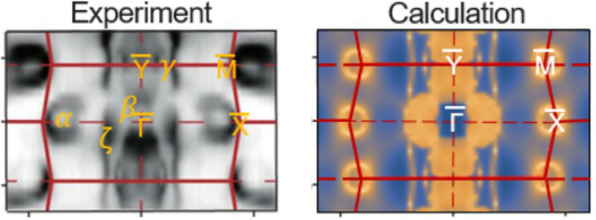

**Supplementary Information:**

The online version contains supplementary material available at 10.1186/s40580-024-00457-y.

## Introduction

The topological classifications of electronic structure have led to a significant discovery of topology-driven phenomena in condensed matter physics. In many of these topological materials, the non-trivial topology is manifested with the surface states, such as linear band crossings protected by time-reversal symmetry in topological insulators and Fermi arcs in Weyl semimetals, giving rise to the gapless metallic states. These properties are determined by bulk wavefunctions over the whole Brillouin zone with discrete topological indices.

Various experimental probes have been used to detect nontrivial band topologies. Angle-resolved photoemission spectroscopy (ARPES) offers a direct method for detecting surface states and their dependence on surface termination [1–6]. Furthermore, transport measurements can be employed to identify topological surface states, with characteristic features such as low-temperature resistivity plateaus and negative magnetoresistance [7–15]. The angular dependence of the quantum oscillation measurements is also used to detect surface states, providing insights into the dimensionality of the states around the Fermi energy [9, 11, 15, 16].

In the case that both bulk and topological surface states exist around the Fermi energy, a multifaceted analysis is required to unambiguously determine the topology of the band structures since both states contribute to the response from the external perturbation. In this case, the ARPES measurements combined with first-principles calculations could be an effective strategy in which the theoretical band structures can be used as a guide to distinguish the surface and bulk electronic structures.

Recently discovered TaSb_2_ exhibits intriguing transport properties, including resistivity plateaus, negative magnetoresistance (MR), extremely large MR (XMR), and non-trivial Berry phase in Shubnikov-de-Hass (SdH) oscillations, indicating the bulk states non-zero Berry curvatures. It is argued that the unusual magnetoresistance is due to the small electron and hole pockets or from magnetic field-induced Weyl points [9, 16]. The first-principles density functional theory (DFT) calculations show the nodal lines, which are gapped in the presence of the spin–orbit coupling (SOC). The calculated topological indices predict the weak topological insulating phase where there are bulk electron and hole pockets crossing the Fermi energy [17], resulting in the topological semimetallic phase. However, there has been no direct observation of the surface states that confirms the proposed topological insulating phase where the topological surface states give rise to the low-temperature resistivity plateaus. This calls for a systematic study of the electronic band structures of TaSb_2_.

In this paper, we investigate the electronic band structure of TaSb_2_ using ARPES and DFT calculations. Our analyses reveal bulk and surface bands in TaSb_2_, providing direct evidence for the existence of the topological surface states. Particularly, most bands near the Fermi level are identified as surface states, while bulk bands are located at relatively higher binding energies (*E-E*_*F*_ < − 0.5 eV, where *E*_*F*_ is Fermi energy), indicating clear topological properties of TaSb_2_ that govern the unique transport phenomena such as the resistivity plateau and XMR. Our study delivers comprehensive investigations into the electronic structures of a complex topological quantum material, providing essential insights into the close correlation between the electronic band structure and transport properties for future technological applications [3, 18–23].

## Results and discussions

### Electron band structures of TaSb_2_

**Fig. 1:**
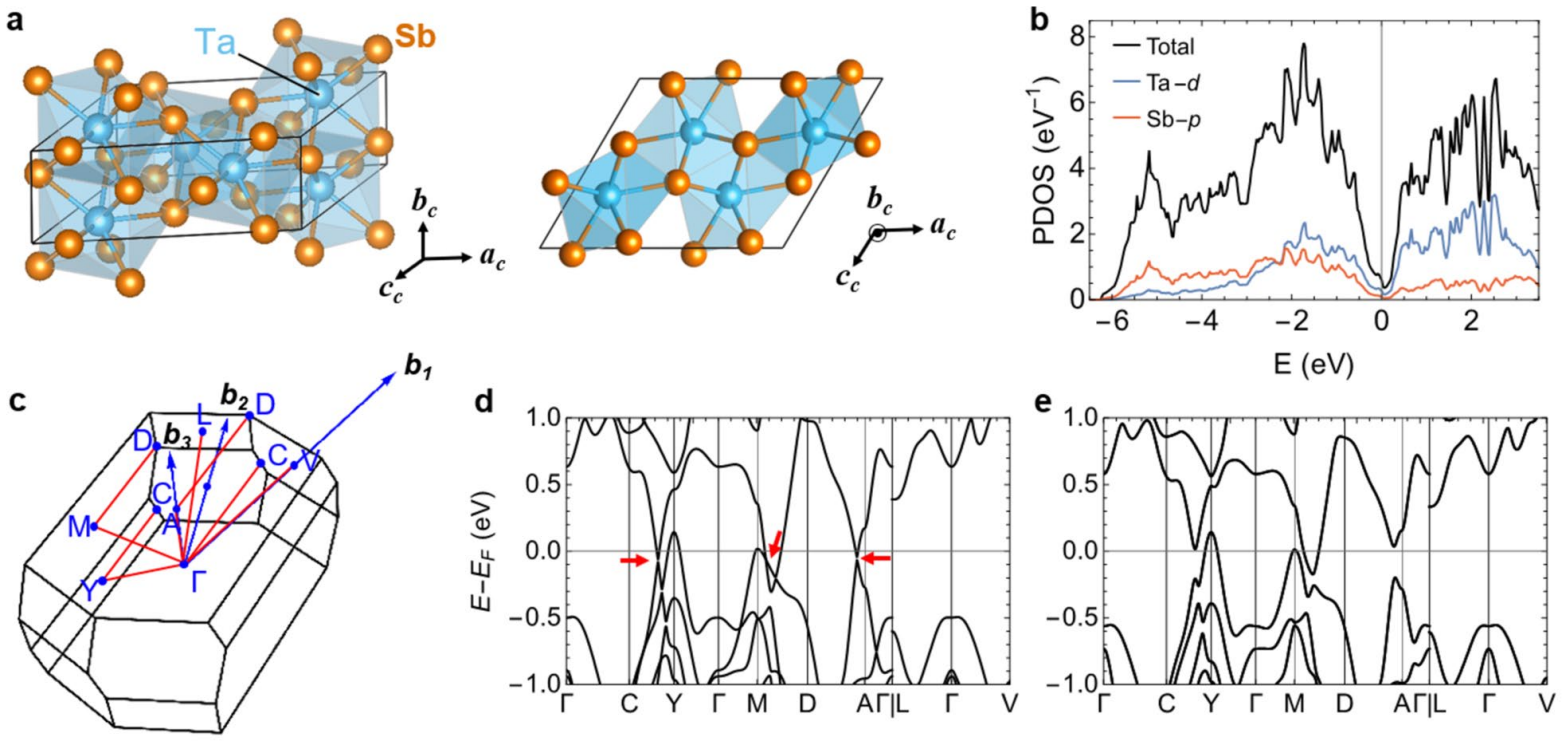
Atomic configuration and electronic structures of TaSb_2_. **a** Perspective and top view of the atomic configurations. The lattice vectors with subscript *c* correspond to the conventional unit cell. **b** The Ta-*d* and Sb-*p* orbital-projected DOS, along with the total DOS. **c** High symmetry points in the Brillouin zone where ***b***_***1***_, ***b***_***2***_, and ***b***_***3***_ are the reciprocal lattice vectors of the primitive unit cell. **d** Band structures without spin–orbit coupling (SOC) along the high-symmetry lines shown with the red solid lines in (**c**). **e** Band structures with SOC

The atomic structure of bulk TaSb_2_ is presented in Fig. 1a. It has a monoclinic unit cell with space group *C2/m* (No. 12), in which each Ta site is surrounded by eight Sb atoms. The calculated lattice constants are *a*_*c*_ = 10.354 Å, *b*_*c*_ = 3.700 Å, and *c*_*c*_ = 8.384 Å in good agreement with experimental data (see SM for detail) [9]. The frontier orbitals around the Fermi energy (*E*_*F*_) are Ta-*d* and Sb-*p* with substantial hybridization between them, as shown in the partial density of states (PDOS) in Fig. 1b. We find a V-shaped density of states around the Fermi energy with a small but finite value at the Fermi energy, consistent with the previous report and metallic transport behavior [9, 17]. The finite density of states at the Fermi energy consists of small electron and hole pockets with band crossings observed along high symmetry lines, as in Fig. 1d, in accordance with the previous DFT calculations [9, 17]. Upon the inclusion of the SOC, the band crossings are fully gapped, but there are electron and hole pockets crossing the Fermi energy, showing a semimetallic ground state.

**Fig. 2:**
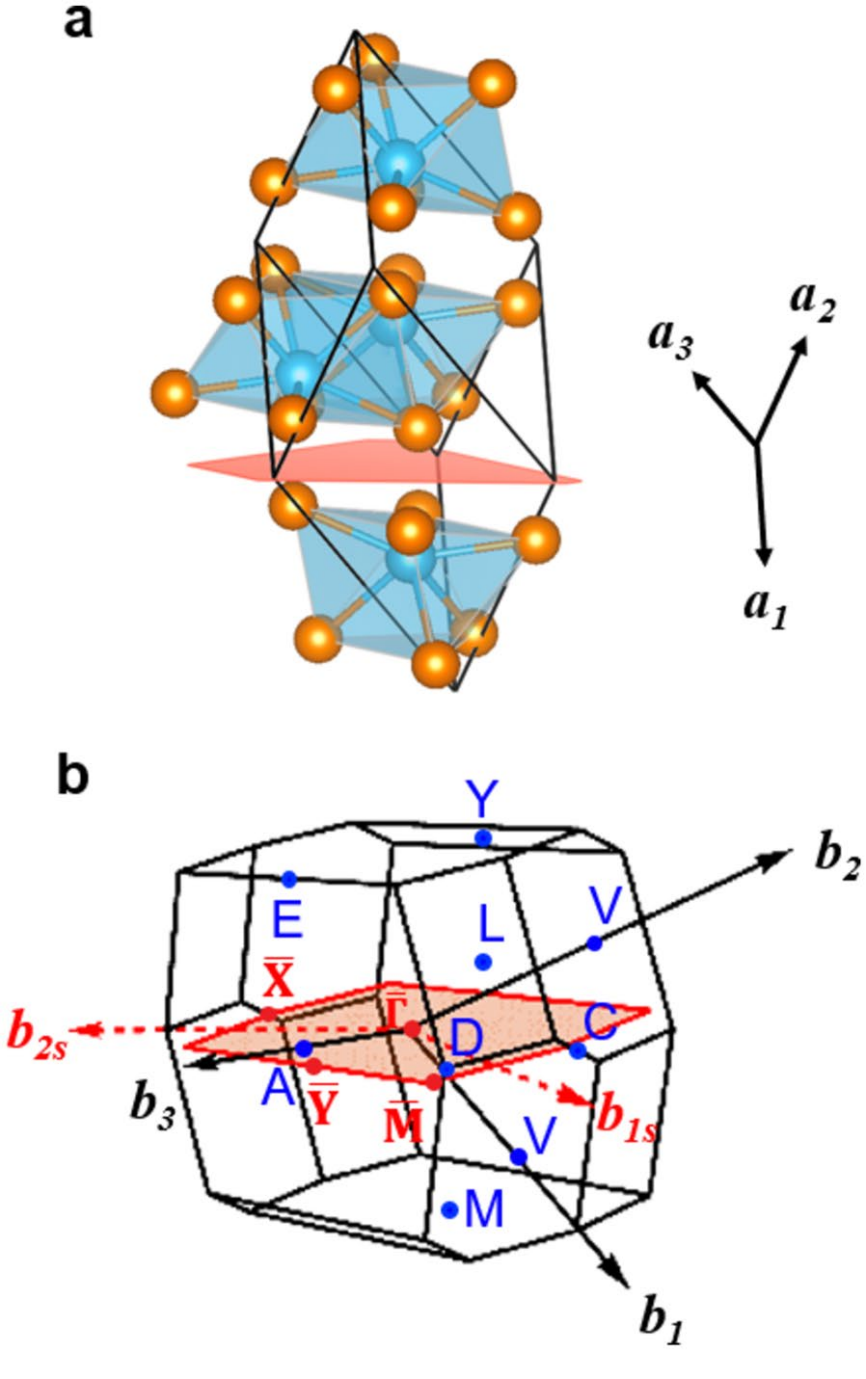
The cleaved atomic plane and surface Brillouin zone. **a** Primitive unit cell with $(1\overline{1 }\overline{1 })$ plane shown as a red-colored area. **b** The surface Brillouin zone (BZ) corresponding to the $(1\overline{1 }\overline{1 })$ plane, denoted as a red-colored area with newly defined high symmetry points, $\overline{\Gamma  }$, $\overline{\text{X} }$, $\overline{\text{Y} }$, and $\overline{\text{M} }$. The red dotted arrows (***b***_***1s***_ and ***b***_***2s***_) are the reciprocal lattice vectors of the surface BZ

Figure 2 shows the natural cleavage plane measured by ARPES. We find that the $(1\overline{1 }\overline{1 })$ plane with respect to the primitive lattice vectors are parallel to the layered atomic planes obtained by disconnecting one of the eight Ta-Sb bonds that has the largest distance (3.01 Å). The corresponding surface Brillouin zone (BZ) is in an elongated hexagonal shape. Moreover, the surface BZ has matching periodicity compared with the surface bands measured by ARPES. Therefore, the $(1\overline{1 }\overline{1 })$ plane can be considered as the cleavage plane, consistent with the distance between the Ta and Sb ions. We note that the identification of this unusual cleavage plane is challenging, as it requires a meticulous comparison between the periodicity of the surface band structures and those obtained by considering all possible cleavage planes. Our successful identification of the cleavage plane enables further analysis of the electronic band structure related to the topological nature.

### Topological surface states of TaSb_2_

**Fig. 3:**
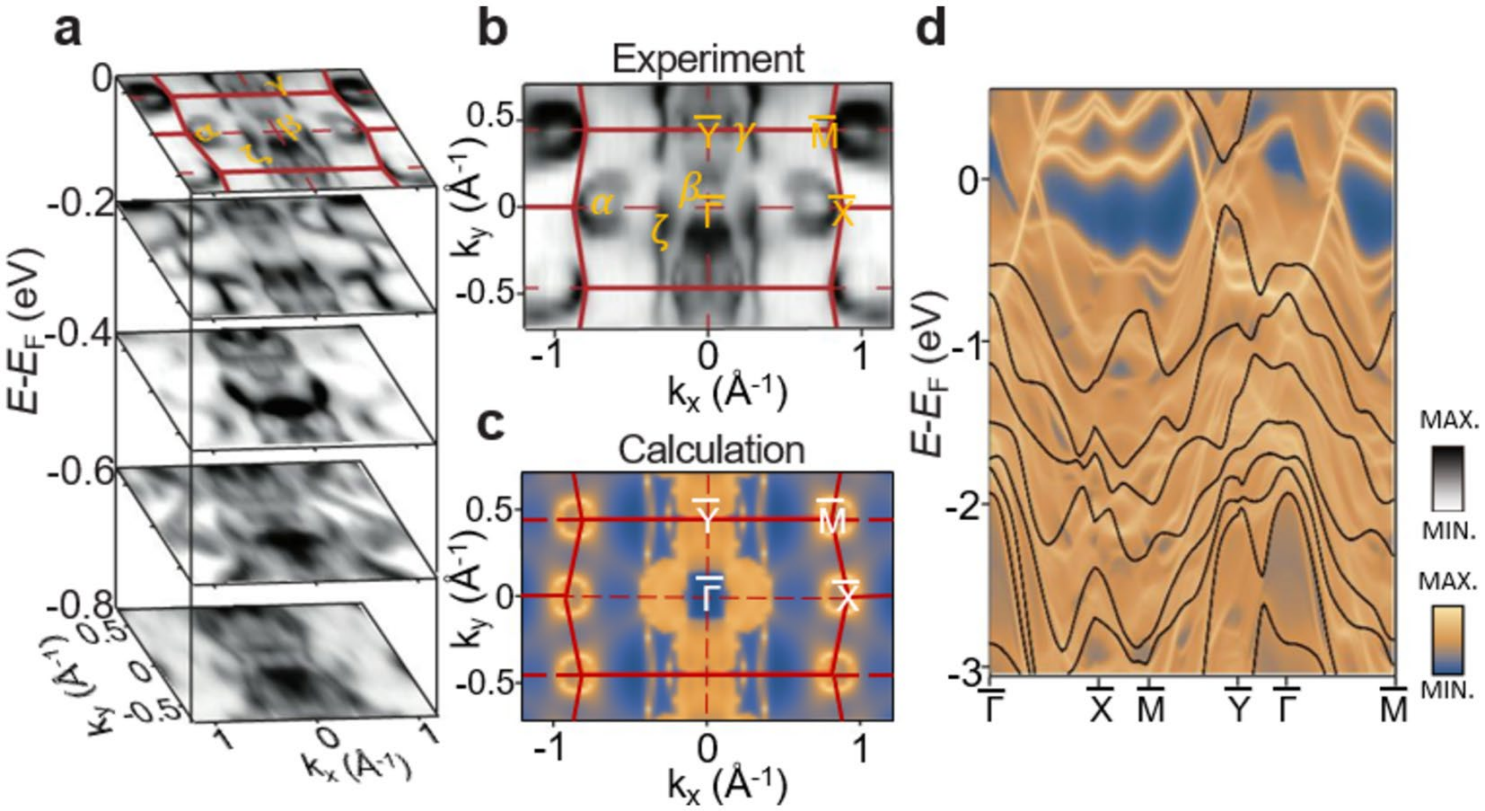
Electronic band structure of TaSb_2_. **a** Constant energy contours in the energy range from *E-E*_*F*_ = 0 eV to − 0.8 eV. Experimental (**b**) and calculated (**c**) Fermi surface (FS) with *hv* = 55 eV. Red lines denote the surface Brillouin zone (SBZ) with high-symmetry direction labeled. The band energies are shifted to the higher binding energy of about 20 meV for better comparison. **d** Calculated band structure with bulk (black solid line) and surface bands (blue to yellow color scale)

To investigate the electronic band structure of TaSb_2_, we performed ARPES measurements and DFT calculations on TaSb_2_ single crystals. ARPES intensity plots of the constant energy contours were stacked with energy values ranging from 0, − 0.2, − 0.4, − 0.6, and − 0.8 eV, respectively (Fig. 3a). The bands at *E-E*_*F*_ = 0 eV were observed, confirming the metallic property of TaSb_2_, consistent with other transport results (Fig. 3b) [9, 16]. The electron-like pocket (α) at the X point disappears with emergence of a wave-shaped bands as binding energy increases (Fig. 3a). The ripple-shaped band (ζ) elongates along the $\overline{\Gamma  }$-$\overline{\text{Y} }$ direction, exhibiting 1D-like chain structures across all stacked binding energies. This feature implies the presence of an open Fermi surface feature, suggesting that the open-orbit fermiology may be a contributing factor to the XMR [24, 25]. The experimental Fermi surface (Fig. 3b), exhibits electron (α) and hole (β)-like pockets, ripple-shaped features (ζ), and single dot points (γ).

From the DFT calculations showing both the surface and bulk band structures, we obtained similar Fermi surface features (Fig. 3c) to the ARPES result (Fig. 3b): open pockets at the $\overline{\text{X} }$ and $\overline{\text{M} }$ points, four closed pockets near the $\overline{\Gamma  }$ point, and a long ripple-shaped feature along the $\overline{\Gamma  }$-$\overline{\text{Y} }$ high symmetry direction, which shows a quasi-1D like shape. The open electron pockets (α) at the $\overline{\text{X} }$ points from DFT results are centered at the $\overline{\text{X} }$ points, whereas they are slightly closer to the $\overline{\Gamma  }$ points in ARPES results. With this exception noted, the overall band structures are in agreement with both DFT and ARPES results. We note that most of the bands near the Fermi energy in the SBZ are derived from the surface states, as the bulk states are mostly pushed into the higher binding energy about 20 meV below the *E*_*F*_ for this particular natural cleavage plane. These surface states are from the weak topological insulating phase of TaSb_2_ consistent with the calculated *Z*_*2*_ classification of (0; 111), where the topological surface states emerge with the band inversion induced by gapped band crossing by SOC, consistent with the previous reports [17].

**Fig. 4:**
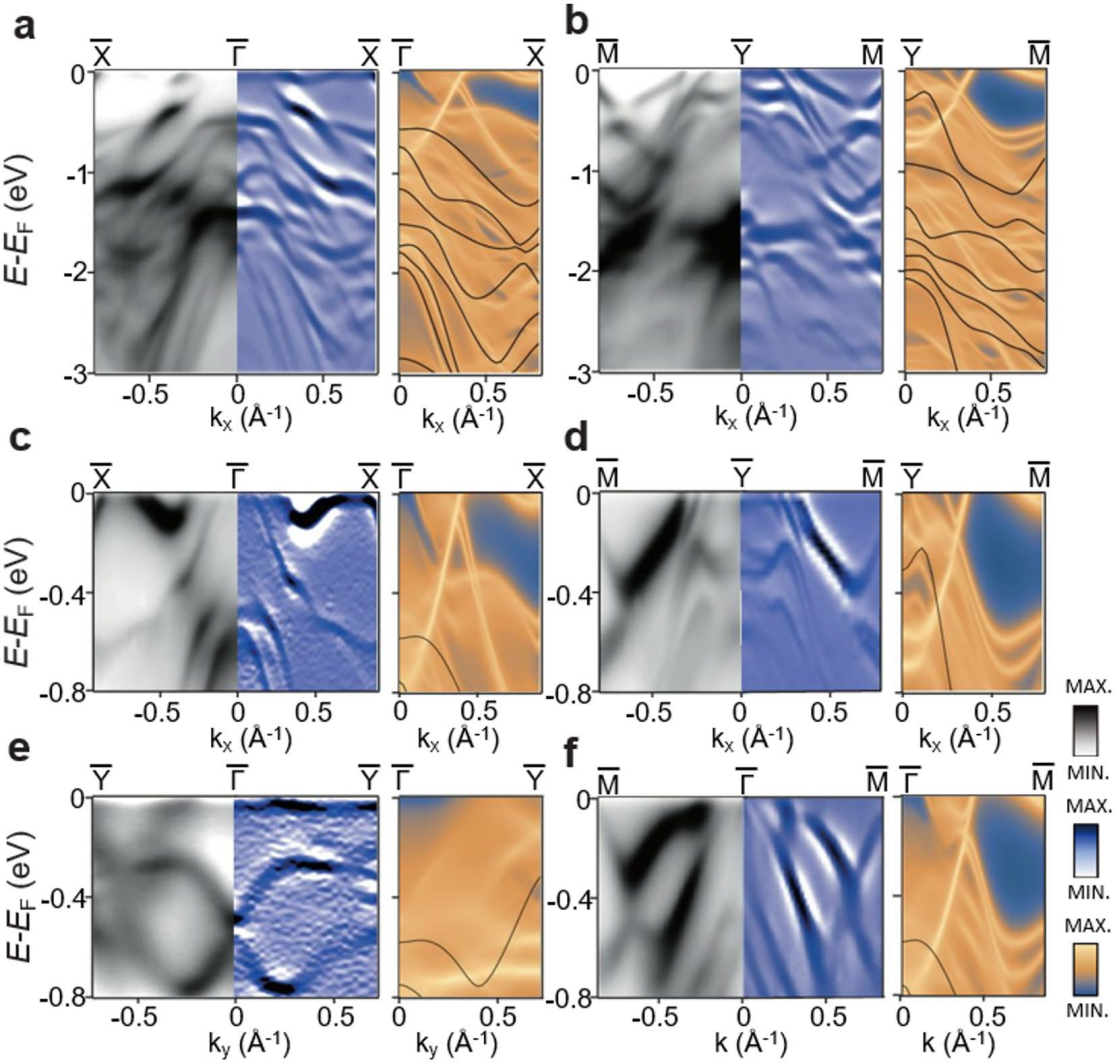
Experimental and theoretical electronic band structures of TaSb_2_. **a**–**f** ARPES intensity plots taken at *hv* = 55 eV (left), corresponding second-derivatives ARPES spectra for enhanced visibility (middle), and calculated band structure (right) along high symmetry directions, $\overline{\Gamma  }$-$\overline{\text{X} }$ (**a**, **c**), $\overline{\text{Y} }$-$\overline{\text{M} }$ (**b**, **d**), $\overline{\Gamma  }$-$\overline{\text{Y} }$ (**e**), and $\overline{\Gamma  }$-$\overline{\text{M} }$ (**f**). In the calculated band structure (right panels), black solid lines represent bulk states, while yellow dispersions (blue to yellow color scale) denote surface bands

We further investigate the experimental electronic band structures of TaSb_2_ along various high symmetry directions, comparing them to the DFT calculations. In Fig. 4, calculated bulk band structures with SOC are presented in black solid lines. They exist mostly away from the Fermi level in higher binding energy in this particular cleavage plane $(1\overline{1 }\overline{1 })$. SOC separates the valence and conduction bands, leading to no band crossing points for the bulk bands, thereby classifying TaSb_2_ as a topological material with weak topological invariants, which is consistent with the previous report [26]. All surface bands, except for the $\overline{\Gamma  }$-$\overline{\text{Y} }$ direction, exhibit band crossings that create nodal lines in line with the previous report [17]. However, bulk bands with SOC gap out the nodal lines and separate the valence and conduction bands. Consequently, TaSb_2_ exhibits nearly compensated semimetal behavior and universally possesses surface states near Fermi level, which leads to TaSb_2_ as a topological insulator [17, 26].

### Topological nature of TaSb_2_

Transition-metal dipnictides, *TPn*_2_ (*T* = Nb, Ta and *Pn* = Sb, As), exhibit unique topological characters. They are weak topological insulators in zero magnetic fields, but under external magnetic fields, can be categorized as Type-II Weyl materials [27]. Typically, it is suggested that the resistivity plateau observed in these materials arises from the competition between the insulating bulk state and metallic surface states. The initial increase in conductivity with decreasing temperature is followed by a resistivity plateau where the conductance of the metallic surface state saturates the resistivity of the insulating bulk state. On the other hand, recent research suggests that classical magneto-resistance theories offer an alternative explanation for these resistivity plateaus observed in materials, without necessarily relying on topological surface states. This alternative explanation considers factors such as impurity scattering, field induced metal–insulator transition, electron–phonon interactions, and electron–electron interactions [11–16].

TaSb_2_ exhibits various interesting transport properties, including positive extreme magnetoresistance (XMR) and high mobility. In the low-temperature regime, it demonstrates both the negative MR and resistivity plateau when the applied field is parallel and perpendicular to the current, respectively [9].

There has been considerable interest in understanding the microscopic mechanisms of XMR and identifying novel XMR materials, such as transition-metal dipnictides (TmPn_2_) MoAs_2_, and W_2_As_3_ [12, 13, 28–30]. Proposed mechanisms to explain XMR include nontrivial band topology, electron–hole compensation, open-orbit Fermi surface (FS) topology, and forbidden backscattering at zero field. In our analysis of the electronic band structure, we observed a distinct non-closing band feature in the FS (Fig. 3a, b), a characteristic consistently present in XMR semimetals of the TmPn_2_ family with the C12/m1 space group. This finding suggests that open-orbit fermiology, together with electron–hole compensation, may play a key role in the XMR behavior of TmPn_2_ materials, including TaSb_2_ [24, 25, 31].

The resistivity plateau observed in TaSb_2_ at low temperatures is a result of a magnetic field-induced resistivity plateau with the broken time-reversal symmetry. This differs from the typical resistivity plateau in topological insulators with time-reversal symmetry, such as NbSb_2_, NbAs_2_, TaAs_2_, and WTe_2_ [15, 28, 31–34]. These properties are attributed to field-induced metal–insulator transition or Kohler’s rule. On the other hand, calculations of the bulk electronic band structure suggest that TaSb_2_ has weak topological properties, potentially leading to the presence of surface states contributing to the observed resistivity plateau [17]. In addition, due to the presence of electron and hole pockets at the Fermi level, this resistivity plateau cannot be elucidated by Kohler’s rule based on single scattering process. Given that most of the transport measurements are done along the [110] direction that is included in the $(1\overline{1 }\overline{1 })$ cleavage plane, the observed numerous surface states near the Fermi surface need also be considered as a major source of the resistivity plateau in addition to the contribution from the compensating bulk electron and hole pockets. We note that our theoretical calculation of another (001) cleavage plane containing the [110] direction show substantial surface states (See SM for details), supporting the robust surface state contributions in the transport along the [110] direction.

## Conclusions

In conclusion, our investigation into the electronic band structure of TaSb_2_ has provided valuable insights into its topological nature and unique transport phenomena. Through a combination of ARPES and DFT calculations, we have identified both bulk and surface bands in TaSb_2_, offering direct evidence of its topological properties. Our results imply that the presence of an open Fermi surface may be a shared characteristic in XMR materials with the C12/m1 space group, potentially working in synergy with electron–hole compensation to elucidate the origin of the XMR effect. In addition, a significant proportion of the bands near the Fermi level are identified as surface states, while bulk bands are situated at relatively higher binding energies. This observation underscores the clear topological properties of TaSb_2_, which can be a key factor in understanding unique transport phenomena such as resistivity plateau. While classical magneto-resistance theories offer alternative explanations for resistivity plateaus, our investigation stresses the critical role of topological surface states in shaping the transport properties of TaSb_2_, suggesting avenues for further exploration of its topological properties.

## Methods

### Single crystal growth

Single crystals of TaSb_2_ were synthesized using chemical vapor transport methods as described previously [9, 16, 35, 36].

### ARPES measurement

ARPES measurements were performed at the HERS endstation of the Beamline 10.0.1, Advanced Light Source, Lawrence Berkeley National Laboratory. The ARPES system is equipped with a Scienta R4000 electron analyzer and has base pressure 3 × 10^–11^ Torr. The photon energy was set at 55 eV with energy and angular resolution of 25 meV and 0.1 degree. Measurements were made at 15 K.

### First-principles calculations

First-principles DFT calculations were performed using the Vienna ab initio simulation package (VASP) [37, 38]. The generalized gradient approximation with Perdew–Burke–Ernzerhof parameterizations [39] was used for the exchange–correlation functional. The projector augmented wave method [40] was used with an energy cut-off of 500 eV. The Γ-point centered 8 × 8 × 5 k-point grid was used. Convergence was reached if the consecutive energy difference was less than 10^–6^ eV. The atomic structures were relaxed with a force threshold of 0.001 eV Å^–1^. For the calculation of the surface band structures and the *Z*_*2*_ index set, Wannier90 code [41] and WannierTools were used [42].

## Supplementary Information

Supplementary Material 1: Table S1. Comparison between the calculated and experimental lattice parameters. Figure S1. *k*_z_ dispersion of TaSb_2_. Figure S2. The cleaved planes parallel to the b_c_ direction and corresponding surface BZs.

## Data Availability

The data generated during the current study are available from the corresponding authors upon reasonable request.

## Abbreviations

ARPES: Angle-resolved photoemission spectroscopy
MR: Magnetoresistance
XMR: Extremely large magnetoresistance
SdH: Shubnikov-de-Hass
DFT: Density functional theory
SOC: Spin–orbit coupling
E_F_: Fermi energy
PDOS: Partial density of states
BZ: Brillouin zone
SBZ: Surface Brillouin zone
XMR: Extreme magnetoresistance
VSAP: Vienna ab initio simulation package
