# Supplementary material for: Topological surface states of semimetal TaSb2
Source: Nano Converg. 2024 Dec 2;11:50. doi: 10.1186/s40580-024-00457-y (PMC11612110; doi:10.1186/s40580-024-00457-y)
Supplement: Supplementary file 1 — Supplementary Material 1: Table S1. Comparison between the calculated and experimental lattice parameters. Figure S1. kz dispersion of TaSb2. Figure S2. The cleaved planes parallel to the bc direction and corresponding surface BZs. [file 40580_2024_457_MOESM1_ESM.docx]

**Supplementary Materials for**

**Topological Surface States of Semimetal TaSb_2_**

Ji-Eun Lee^1,2,3^, Yu Liu^4,5^, Jinwoong Hwang^6^, Choongyu Hwang^7^, Cedomir Petrovic^8,5^, Se Young Park^9,10 *^, Hyejin Ryu^2,*^, and Sung-Kwan Mo^1,*^

*^1^Advanced Light Source, Lawrence Berkeley National Laboratory, Berkeley, CA 94720, USA*

*^2^Center for Spintronics, Korea Institute of Science and Technology (KIST), Seoul 02792, South Korea*

*^3^Max Planck POSTECH Center for Complex Phase Materials, Pohang University of Science and Technology, Pohang 37673, South Korea*

*^4^Center for Correlated Matter and School of Physics, Zhejiang University, Hangzhou 310058, China*

*^5^Condensed Matter Physics and Materials Science Department, Brookhaven National Laboratory, Upton, New York 11973, United States*

*^6^Department of Physics and Institute of Quantum Convergence Technology, Kangwon National University, Chuncheon 24341, South Korea*

*^7^Department of Physics, Pusan National University, Busan 46241, South Korea*

*^8^Shanghai Key Laboratory of Material Frontiers Research in Extreme Environments (MFree), Shanghai Advanced Research in Physical Sciences (SHARPS), Pudong, Shanghai 201203, China*

*^9^Department of Physics and Origin of Matter and Evolution of Galaxies (OMEG) Institute, Soongsil University, Seoul 06978, South Korea*

*^10^Integrative Institute of Basic Sciences, Soongsil University, Seoul, 06978, South Korea*

**Table S1 | Comparison between the calculated and experimental lattice parameters.**

|  | ***a_c_* (Å)** | ***b_c_* (Å)** | ***c_c_* (Å)** | **α (deg.)** | **β (deg.)** | **γ (deg.)** | **Vol. Å^3^)** |
| --- | --- | --- | --- | --- | --- | --- | --- |
| **Calculated** | 10.354 | 3.7 | 8.384 | 90 | 120.546 | 90 | 276.6 |
| **Exprimental^1^** | 10.222 | 3.645 | 8.292 | 90 | 120.39 | 90 | 266.5 |
| **Error (%)** | 1.3 | 1.5 | 1.1 | 0.0 | 0.1 | 0.0 | 3.8 |

**
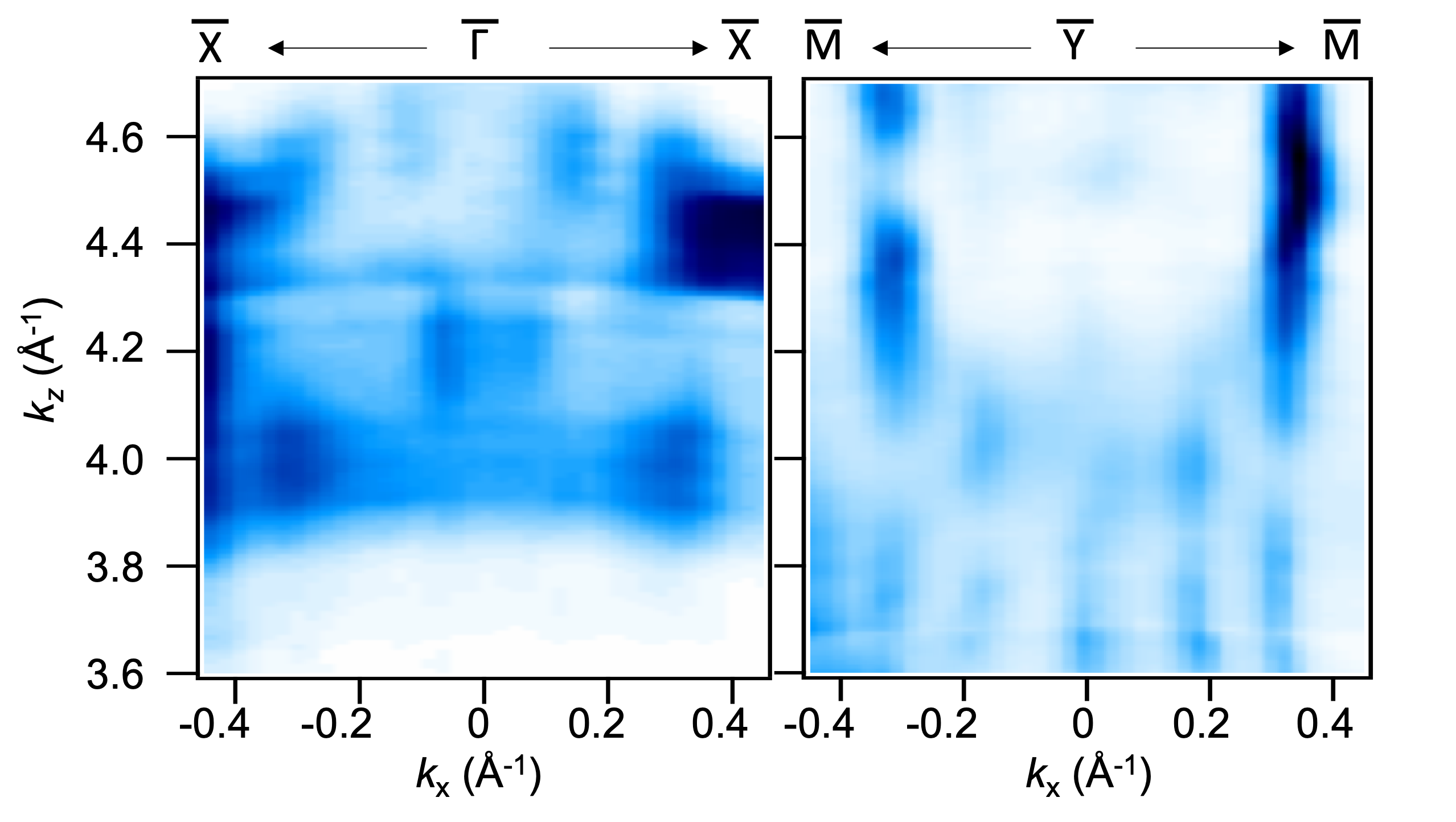
**

**Figure S1 | *k*_z_ dispersion of TaSb_2._** The photon energy dependence (k_x_-k_z_ dispersions) of the electronic band structures of TaSb_2_ along the Γ-X and Y-M directions at the Fermi level exhibits minimal dispersion along the k_z_ direction in the ARPES spectra, a characteristic feature typically associated with the surface states. This observation suggests that the surface states dominate the electronic contributions near the Fermi level.


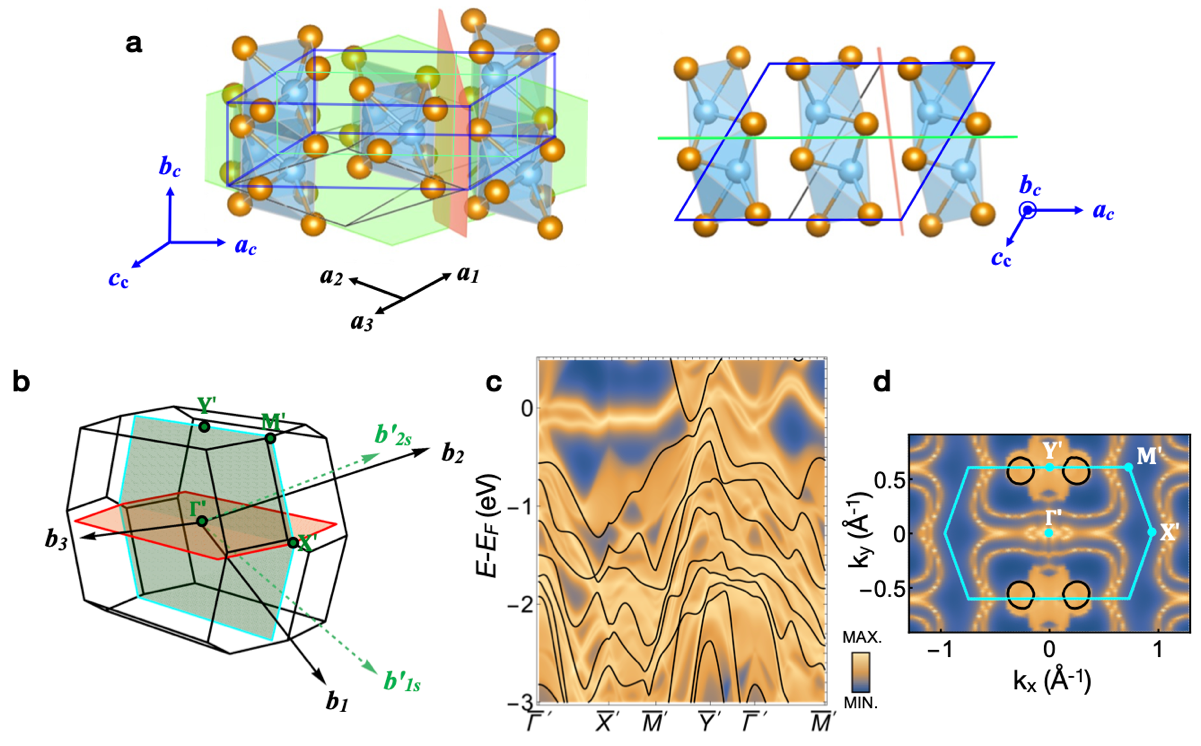


**Figure S2 |** **The cleaved planes parallel to the b_c_ direction and corresponding surface BZs.** **a.** The primitive (black solid lines) and conventional (blue solid lines) unit cells. The red- and green-colored areas correspond to the $(1\bar{1}\bar{1})$ and (001) cleavage planes, respectively. These two planes are parallel to ***b_c_*** (or [110] direction of the primitive cell). The figure on the right shows the top view. **b**. The surface BZs of $(1\bar{1}\bar{1})$and (001) planes, denoted as a red- and green-colored area, respectively. The high symmetry points of (001) surface BZ are marked with $\bar{\Gamma}'$, $\bar{X}'$, $\bar{Y}'$, and $\bar{M}'$ symbols. The green dotted arrows (***b'_1s_*** and ***b'_2s_***) are the reciprocal lattice vectors of the (001) surface BZ. **c-d**. Calculated band structures **(c)** and Fermi surfaces **(d)**, showing bulk (black solid line) and surface (blue to yellow color scale) states. The area enclosed by cyan lines is the (001) surface BZ. For the Fermi surfaces, the band energies are shifted to the higher binding energy of 20 meV to be consistent with the Fermi surface in Fig. 3 **c**.

**References**

S1. Li, Y., Li, L., Wang, J., Wang, T., Xu, X. et al. Resistivity plateau and negative magnetoresistance in the topological semimetal TaSb2. Phys. Rev. B 94, 121115 (2016). 10.1103/PhysRevB.94.121115
